# Trace Metal Availability Affects Greenhouse Gas Emissions and Microbial Functional Group Abundance in Freshwater Wetland Sediments

**DOI:** 10.3389/fmicb.2020.560861

**Published:** 2020-09-30

**Authors:** Georgios Giannopoulos, Katherine R. Hartop, Bonnie L. Brown, Bongkeun Song, Lars Elsgaard, Rima B. Franklin

**Affiliations:** ^1^Department of Biology, Virginia Commonwealth University, Richmond, VA, United States; ^2^Department of Biological Sciences, University of New Hampshire, Durham, NH, United States; ^3^Department of Biological Sciences, Virginia Institute of Marine Science, College of William & Mary, Gloucester Point, VA, United States; ^4^Department of Agroecology, Aarhus University, Tjele, Denmark

**Keywords:** denitrification, DNRA, carbon mineralization, wetland microbes, trace metals

## Abstract

We investigated the effects of trace metal additions on microbial nitrogen (N) and carbon (C) cycling using freshwater wetland sediment microcosms amended with micromolar concentrations of copper (Cu), molybdenum (Mo), iron (Fe), and all combinations thereof. In addition to monitoring inorganic N transformations (NO_3_^–^, NO_2_^–^, N_2_O, NH_4_^+^) and carbon mineralization (CO_2_, CH_4_), we tracked changes in functional gene abundance associated with denitrification (*nirS*, *nirK*, *nosZ*), dissimilatory nitrate reduction to ammonium (DNRA; *nrfA*), and methanogenesis (*mcrA*). With regards to N cycling, greater availability of Cu led to more complete denitrification (i.e., less N_2_O accumulation) and a higher abundance of the *nirK* and *nosZ* genes, which encode for Cu-dependent reductases. In contrast, we found sparse biochemical evidence of DNRA activity and no consistent effect of the trace metal additions on *nrfA* gene abundance. With regards to C mineralization, CO_2_ production was unaffected, but the amendments stimulated net CH_4_ production and Mo additions led to increased *mcrA* gene abundance. These findings demonstrate that trace metal effects on sediment microbial physiology can impact community-level function. We observed direct and indirect effects on both N and C biogeochemistry that resulted in increased production of greenhouse gasses, which may have been mediated through the documented changes in microbial community composition and shifts in functional group abundance. Overall, this work supports a more nuanced consideration of metal effects on environmental microbial communities that recognizes the key role that metal limitation plays in microbial physiology.

## Introduction

Wetland microbes are important for removing anthropogenic pollutants from surface waters, effectively preventing the contaminants from entering downstream coastal and marine ecosystems. Human-derived industrial nitrogen (N) input to the environment has been estimated to 150 Tg N y^–1^ (Schlesinger, 2009), and is of particular concern because it is linked to water quality degradation, eutrophication, and increased emissions of nitrous oxide (N_2_O), which is a strong greenhouse gas (GHG) that also contributes to ozone depletion (Ravishankara et al., 2009; IPCC, 2013). Biological production of N _2_O is mainly through denitrification, nitrification, and nitrifier-dentrification; in addition, some denitrifying microbes can remove N_2_O by reducing it to inert dinitrogen (N_2_). Annually, ∼10% of the human-derived N input is returned to the atmosphere as N_2_ via denitrification from wetlands and terrestrial ecosystems globally (Schlesinger, 2009). Wetlands are equally important for their role in carbon (C) cycling. Despite their relatively small coverage (∼10% of global land area), these ecosystems store large amounts of organic C and emit considerable amounts of methane (CH_4_) at an estimated rate of 144 Tg CH_4_ y^–1^ (IPCC, 2014). These net CH_4_ emissions represent a balance between the microbial processes of methanogenesis and methanotrophy.

Microbial production and consumption of N_2_O and CH_4_ is catalyzed by oxidoreductases that utilize metal co-factors such as molybdenum (Mo), copper (Cu), and iron (Fe) (Glass and Orphan, 2012). For denitrification, the pathway includes the reduction of nitrate (NO_3_^–^) to nitrite (NO_2_^–^), catalyzed by a periplasmic or membrane bound Mo nitrate reductase (NAR) (Schwarz et al., 2009), and the further reduction of NO_2_^–^ to nitric oxide (NO) by either a heme-Fe cytochrome c*d*_1_ or a Cu co-factor nitrite reductase (NIR), expressed by the genes *nirS* and *nirK*, respectively (Adman et al., 1995; Einsle et al., 1999). NO is further reduced to N_2_O by nitric oxide reductase (NOR), which contains a heme-Fe co-factor (Shiro et al., 2012). Finally, N_2_O is reduced by a Cu-containing nitrous oxide reductase (NOS) (Rosenzweig, 2000). Similar metal complexes are key in methane cycling. For example, Fe-, Ni-, and Zn-dependent ferredoxins and dehydrogenases catalyze the early steps of methanogenesis, and all methanogens utilize a common Ni methyl-coenzyme M reductase (MCR; *mcrA*) to catalyze the rate-limiting and final step of CH_4_ production (Glass and Orphan, 2012; Wongnate and Ragsdale, 2015). Cu also has an integral role in CH_4_ oxidation. For example, aerobic methanotrophs scavenge Cu with methanobactin, a high-affinity chaperone (Chang et al., 2018), for use in Cu-dependent CH_4_ monoxygenases. Some anaerobic methanotrophs, such as members of the NC10 phylum (e.g., *Methylomirabilis oxyfera*), utilize a Cu-dependent particulate methane monoxygenase (pMMO) and an Fe-rich *cd*_1_ nitrite reductase to couple CH_4_ oxidation to denitrification via nitrite-dependent methane oxidation (N-DAMO) (Deutzmann et al., 2014; Cheng et al., 2019). Because of these sorts of interconnected pathways and processes, the abundance and bioavailability of trace metals in the environment can impact N and C biogeochemistry and exert a control on associated GHG emissions.

Although extensive research has considered the relative contribution of key environmental factors (e.g., pH, NO_3_^–^, NO_2_^–^, O_2_, and C/N ratio) in regulating the microbial processes associated with N and C biogeochemistry, our understanding of the impact of metal availability is more limited. Metals in the environment have traditionally been seen as unwanted pollutants, and trace metal accumulation has been linked to toxicity and inhibition of ecosystem processes (Samanidou and Papadoyannis, 1992). For example, concentrations exceeding the mg L^–1^ range for Cu, Mo, Fe, Zn, and Pb severely inhibit denitrification in soils, sediments, surface water bodies, and waste waters (Labbé et al., 2003; Magalhães et al., 2007; Liu et al., 2016). However, given the dependence of many microbial enzymes on metal co-factors, it also is possible for the opposite effect to occur – wherein enzyme function is limited due to an inadequate supply of trace metals. This scenario has received limited attention in environmental studies, but is well documented in case studies with model organisms. In fact, *in vitro* studies have shown that lack of Cu, Mo, or Fe severely inhibits denitrification or methane cycling due to the formation of non-functional enzymes typically lacking the respective metal co-factor. For example, in the soil bacteria *Paracoccus denitrificans* and *Pseudomonas stutzeri*, Cu is required to express a functional NOS dimer and reduce N_2_O to N_2_ (Granger and Ward, 2003; Felgate et al., 2012; Black et al., 2016). In methanotrophs oxidizing CH_4_ to methanol (CH_3_OH), the switch between a Cu-dependent pMMO or Fe-dependent soluble MMO (sMMO) is regulated by the availability of each metal (Murrell et al., 2000; Bollinger, 2010).

Despite great progress in understanding metal ecotoxicity and metalloenzyme biochemistry, our knowledge of how trace metal availability affects microbial activity in the environment is generally limited to selected processes such as ammonium (NH_4_^+^), NO_3_^–^, and CO_2_ assimilation in aquatic ecosystems (Twining et al., 2007; Glass et al., 2012; Moore et al., 2013; Romero et al., 2013; Schoffman et al., 2016). A broader understanding of how metal availability regulates microbial processes, especially in soils and sediments, is necessary if we are to fully comprehend environmental controls on ecosystem N and C cycling. In this study, we investigated the effects of trace metal additions on the microbial biogeochemistry of freshwater wetland sediments focusing primarily on NO_3_^–^/NO_2_^–^ reduction and GHG kinetics, and used quantitative polymerase chain reaction (qPCR) to assess changes in the abundance of key microbial functional groups associated with these processes. We hypothesized that greater bioavailability of Mo, Fe, and Cu in the sediments would increase denitrification and that greater Cu availability would reduce N_2_O emissions (i.e., by stimulating NOS functioning). We also predicted that CO_2_ emissions would increase, driven by higher denitrification rates, and CH_4_ production would decrease because stimulated denitrifiers would outcompete methanogens for C substrates under anoxic conditions.

## Materials and Methods

### Sampling and Sediment Properties

Wetland soil was collected from a tidal-fluvial bar deposit in Pamunkey River (Virginia, United States; N 37.557451, W −76.972521) in October 2016. Five samples from the 5–15 cm depth interval (∼350 g each) were collected ∼2 m apart using a PVC core (diameter, 10 cm). Porewater was collected from a small pit, which was emptied and allowed to refill naturally with porewater prior to collection. Samples were kept on ice in a cooler during transport to the lab and stored overnight (4°C) until experimental setup and characterization using standard methods (SSSA, 1996). Total metal content was analyzed according to United States Environmental Protection Agency (EPA) standard methodology at the Soil Testing, Insect ID and Plant Diagnostic Lab, Cooperative Extension of the University of the New Hampshire (Durham, NH, United States), and porewater Fe(II) concentrations were measured spectrophotometrically following Viollier et al. (2000). Sediment and porewater properties are summarized in Table 1. Previous studies at this site had identified these sediments to be conducive of denitrification, dissimilatory nitrate reduction to ammonium (DNRA), and methanogenesis (Berrier, 2019; Dang et al., 2019).

**TABLE 1:** Initial properties of sediment and pore-water.

**Sediment properties**		**Pore-water concentrations**	
Organic matter (%)	25 (±4)	NO_3_^–^ (mM)	0.10 (±0.03)
Gravimetric water content (%)	79 (±6)	NO_2_^–^ (mM)	0.07 (±0.02)
Bulk density (g cm^–3^)	0.18 (±0.05)	SO_4_^2–^ (mM)	0.16 (±0.04)
C/N ratio	11 (±2)	Cl^–^ (mM)	0.4 (±0.1)
pH (1:5_H__2__O_)	6.1 (±0.3)	NH_4_^+^ (mM)	0.3 (±0.1)
Co (mg kg^–1^)	8.3	K^+^ (mM)	0.11 (±0.01)
Cu (mg kg^–1^)	37.8	Mg^2+^ (mM)	0.26 (±0.10)
Fe (g kg^–1^)	13.5	Ca^2+^ (mM)	0.29 (±0.10)
Mo (mg kg^–1^)	5.2	Fe^2+^ (μM)	97.3 (±81.9)
Ni (mg kg^–1^)	16.2		
Zn (mg kg^–1^)	135.6		

*Data are shown as mean ± standard error (*n* = 4) except for total metal content, which was analyzed using a composite sample.*

### Experimental Set-up

After removal of visible root and stone fragments, the five soil samples were combined in equal parts to form a composite sample for subsequent experiments. The soil (1 kg) was amended with 1.5 L of filtered porewater (Cellulose, 10-μm pore-size, Millipore, Burlington, MA, United States) and homogenized using a commercial blender (30 s at max speed) to a final soil-to-liquid ratio of 1:3.5 (dry w/v). Soil slurry aliquots (50 mL) were then transferred to sterile 125-mL glass bottles (Wheaton, Millville, NJ, United States). The bottles were crimped with rubber septa (#224100-180, Weaton, Millville, NJ, United States) and incubated for 3 days in the dark (25°C) to allow residual O_2_ to be consumed. Each bottle then received 250 μL of 2 M KNO_3_, yielding a final nominal NO_3_^–^ concentration of 10 mM, ensuring non-limiting N conditions corresponding to typical NO_3_^–^ concentrations used in DEA protocols (SSSA, 1994; Murray and Knowles, 1999). Next, metal additions were accomplished by dispensing small aliquots (<1 ml total) from aqueous stock solutions of ammonium heptamolybdate [(NH_4_)_6_Mo_7_O_24_], iron sulfate (FeSO_4_), and copper sulfate (CuSO_4_) to generate final slurry concentrations of 28 μM Mo, 74 μM Fe, and 26 μM Cu. Metal additions were based on a preliminary assessment of N_2_O emissions derived by a model denitrifying bacterium, *P. denitrificans*, incubated with different Mo, Fe, and Cu levels (Supplementary Table S1). Seven experimental treatments were established to test the effect of each metal individually (Mo, Fe, or Cu) and in all possible combinations (Mo + Fe, Mo + Cu, Fe + Cu, and Mo + Fe + Cu). In addition, control microcosms were prepared, to which no metals were added. Four replicate microcosms were prepared for each control and treatment. Each bottle was then vortexed briefly (30 s, 6000 rpm), flushed with N_2_ (60 min), and incubated without shaking in the dark (25°C). Gas (5 mL) and slurry supernatant (1.5 mL) samples were collected using a sterile syringe after 0, 6, 12, 24, 48, 72, and 96 h. Withdrawn gas volumes were replaced with equal N_2_ gas volumes. Following the 96-h sampling, bottles were opened in an O_2_-free chamber, and 0.3 g dry weight of soil was removed and immediately frozen (−20°C) for molecular analyses.

### Analytical Techniques and Calculations

Slurry samples were centrifuged (10,000 *g*, 5 min) and the resulting supernatant was filtered (0.22-μm pore size), and stored frozen (−20°C) until the concentrations of NO_3_^–^, NO_2_^–^, sulfate (SO_4_^2–^), and NH_4_^+^ was determined by ion-chromatography (ICS – 5000+, Dionex, Sunnydale, CA, United States) utilizing Eluent Generator Cartridges; 23–45 mM KOH and 20 mM methanesulfonic acid, for anion and cation analysis respectively. Ions were separated through Dionex IonPac AG18/AS18 (2 × 250 mm) and IonPac CG12/CS12 (A – 5 μm, 3 × 150 mm) columns with suppression, AERS 500 and CERS 500 for anion and cation analysis, respectively, and detected by a Dionex CD conductivity sensor.

Gas samples were stored in 3 mL Exetainer vials (Labco, United Kingdom) that previously were flushed with N_2_ and vacuumed with a gas-tight syringe by removing four volumes, i.e., resulting in a vial pressure of ca. 6 kPa. Concentrations of N_2_O, CO_2_, and CH_4_ were determined via gas chromatography (Shimadzu GC-14A using Porapak-N and HayeSep-D and Molecular Sieve MS13 columns and equipped with ECD (N_2_O), TCD (CO_2_), and FID (CH_4_) detectors (Shimadzu, Columbia, MD, United States) as described in Morrissey and Franklin (2015). For each gas, total gas production was determined as the sum of the gas accumulated in the headspace and the gas dissolved in the liquid slurry (e.g., Robertson et al., 1999) using the Ideal gas law, Henry’s gas solubility law, and Bunsen coefficients (at 25°C) of 0.545, 0.032, and 0.772 for N_2_O, CH_4_, and CO_2_, respectively (Felgate et al., 2012; Sander, 2015). To compare overall GHG emissions across treatments, we estimated the combined global warming potential (GWP) of CO_2_, N_2_O, and CH_4_ in terms of CO_2_-equivalents (g CO_2_-eq) using the 100 years GWP factors of 298 for N_2_O and 28 for CH_4_ as calculated on a mass to mass basis (IPCC, 2013).

### Molecular Techniques

DNA was extracted from frozen soil samples (∼0.3 g dry weight) using the DNEasy Kit from Qiagen (Germantown, MD, United States) following the manufacturer’s instructions The retrieved DNA was visualized on a 1.2% agarose gel and quantified using a Nanodrop spectrometer (Thermo Fisher Scientific, Wilmington, DE, United States). Quantitative PCR (qPCR) was used to determine the abundance of microbial groups typically found in wetland sediments: total bacteria (*eub*), nitrite reducers (denitrification: *nirK* and *nirS*; DNRA: *nrfA*), nitrous oxide reducers (*nosZ-clade I* and *II*), and methanogens (*mcrA*). SensiFAST^TM^ SYBR^®^ No-ROX Kit Polymerase 2× mix (Bioline, United Kingdom) was used for all reactions except *nosZ-clade II*, which used SYBR green Go-Taq^®^ qPCR Master Mix (Promega, Madison, WI, United States). Primers were purchased from IDT (Integrative DNA Technologies, Skokie, IL, United States) and the DNA for the standard curves was extracted from isolates obtained from ATCC (American Type Culture Collection, Manassas, VA, United States). Data were collected using a Bio-Rad CFX-384 Real Time System (Bio-Rad, Hercules, CA, United States) and analyzed using CFX Manager software (Ver. 3.1), except *nosZ-clade II* quantification was conducted using QuantStudio 6 Flex (Thermo Fisher Scientific, Wilmington, DE, United States). Primers, qPCR reaction conditions, and efficiencies are summarized in Table 2.

**TABLE 2:** Primers and reaction conditions for qPCR assays.

**Target**	**Primers**	**PCR Mix (15 μL)**	**Standard**	**Reaction Condition & Efficiency**	**References**
Eubacteria (*16S rRNA*)	*eub*338 *eub*518	1.2 ng template, 0.1 μM each primer	*Desulfovibrio desulfuricans* ATCC 27774	95°C for 4 min, then 40 cycles of 30 s at 95°C, 30 s at 55.5°C, and 60 s at 72°C. *E* = 102%, *R^2^* = 0.992.	Fierer et al., 2005
Denitrifiers (*nirS*)	cd3aF R3cd	10 ng template, 0.1 μM each primer	*Paracoccus denitrificans* ATCC 17741	95°C for 4 min, then 50 cycles of 30 s at 95°C, 30 s at 56°C and 60 s at 72°C. *E* = 113%, *R^2^* = 0.993.	Throback et al., 2004
Denitrifiers (*nirK*)	*nirK*q-F *nirK*1040	1.5 ng template, 0.35 μM each primer	*Pseudomonas* sp. ATCC 13867	15 min at 95°C, 9 touchdown cycles of 95°C for 15 s, 68°C for 60 s, and 81.5°C for 30 s (−1°C per cycle for annealing); then 28 cycles of 95°C for 15 s, 60°C for 60 s, and 81.5°C for 30 s. *E* = 116%, *R^2^* = 0.974.	Smith et al., 2007
Denitrifiers (*nosZ clade I*)	*nosZ*1F *nosZ*2F	10 ng template, 1 μM each primer	*Pseudomonas fluorescens* C7R12	15 min at 95°C, 6 cycles of 95°C for 15 s, 67°C for 30 s with a touchdown of −1°C by cycle, 72°C for 30 s, and 80°C for 15 s (acquisition data step); 40 cycles of 95°C for 15 s and 62°C for 15 s, 72°C for 30 s, and 80°C for 15 s; and 1 cycle at 95°C for 15 s and 60°C for 15 s, to 95°C for 15 s. *E* = 92%, *R^2^* = 0.978.	Henry et al., 2006
Denitrifiers (*nosZ clade II*)	*nosZIIF nosZIIR*	10 ng template, 1.5 μM each primer	*Plasmid- nosZII*	15 min at 95°C, 55 cycles of 95°C for 15 s, 54°C for 30 s, 72°C for 30 s, and 80°C for 35 s. *E* = 63%, *R^2^* = 0.990.	Semedo et al., 2018
DNRA (*nrfA*)	*nrfA*6F *nrfA*6R	10 ng template, 0.3 μM each primer	*Escherichia coli* ATCC 11775	50°C for 2 min, 95°C for 8.5 min, and 50 cycles of 20 s at 94°C, 40 s at 54.5°C, and 10 s at 72°C. *E* = 101%, *R^2^* = 0.987.	Takeuchi, 2006
Methanogens (*mcrA*)	*Mlas mcrA*-rev	2 ng template, 0.6 μM *mlas*, 0.7 μM *mcrA*-rev	*Methanococcus voltae* ATCC BAA-1334	95°C for 5 min, then 50 cycles of 20 s at 95°C, 20 s at 59°C, and 45 s at 72°C. *E* = 93%, *R^2^* = 0.991.	Steinberg and Regan, 2009

### Statistical Analyses

Data did not comply with the normality and variance homogeneity assumptions for ANOVA [RStudio (*base*); Shapiro’s test, Bartlett’s test]; therefore, non-parametric Kruskal-Wallis tests with Bonferroni correction were applied to assess differences in the mean of ranks among the treatments [RStudio (*agricolae*)], without any data transformation. For all statistical tests, *p* ≤ 0.05 was considered significant. Summary statistics were calculated with RStudio (*dyplr*) (Boston, MA, United States) and plotted with SigmaPlot 14 (Systat Software Inc., San Jose, CA, United States). Central tendency and measures of dispersion are shown as mean ± standard error (SE) with *n* = 4, unless otherwise specified.

## Results

### The Effect of Trace Metal Additions on NO_3_^–^, NO_2_^–^, and NH_4_^+^ Kinetics

**FIGURE 1:**
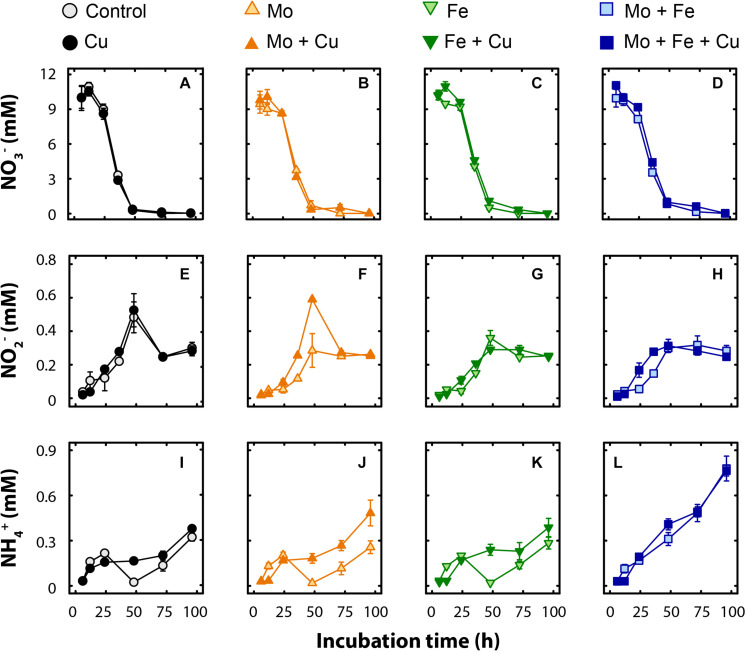
Time course of pore-water concentrations (mM) of nitrate (NO_3_^–^; **A–D**), nitrite (NO_2_^–^; **E–H**), and ammonium (NH_4_^+^; **I–L**) in microcosms amended with molybdate (Mo), iron (Fe), copper (Cu), and combinations thereof. Data are shown as mean ± standard error (*n* = 4).

Kinetics of NO_3_^–^ and NO_2_^–^ transformations in microcosm pore-water were similar across all treatments. Nitrate reduction commenced at the same time for all the treatments (12–24 h, Figure 1), and the NO_3_^–^ pool (10–11 mM) was generally consumed within the first 48 h of the incubation. Nitrite was produced concurrently with NO_3_^–^ depletion and, on average, 0.25 ± 0.02 mM NO_2_^–^ remained across all treatments at the end of the experiment (Figure 1). An increasing trend in pore-water NH_4_^+^ concentration was observed for all treatments during the incubation, and treatments receiving additional Mo + Fe (*p* < 0.01) and Mo + Fe + Cu (*p* < 0.01) had significantly higher NH_4_^+^ than the control at the end of the experiment (Figure 1). On average, 2.7 ± 0.1% and 4.6 ± 0.7% of NO_3_^–^ was recovered as NO_2_^–^ or NH_4_^+^, respectively, at the end of the incubation.

### The Effect of Trace Metal Additions on N_2_O, CH_4_, and CO_2_ Cumulative Gas Kinetics

**FIGURE 2:**
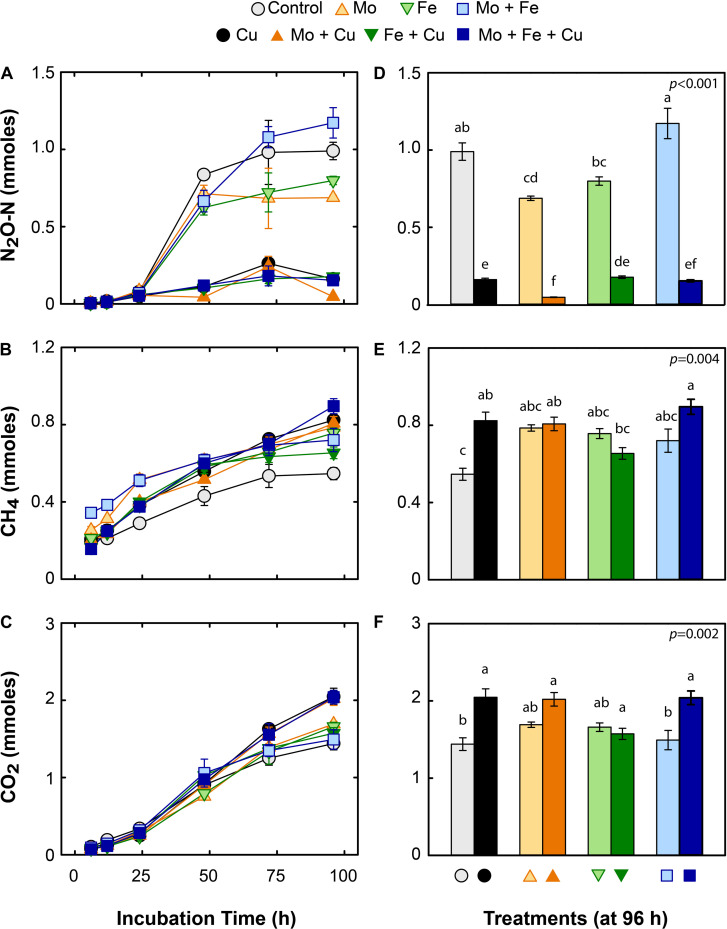
Time course **(A–C)** and final cumulative gas concentrations (96 h; **D–F**) of nitrous oxide (N_2_O; upper panels), methane (CH_4_; middle panels), and carbon dioxide (CO_2_; lower panels) in mmoles per microcosms amended with molybdate (Mo), iron (Fe), copper (Cu), and combinations thereof. Data are shown as mean ± standard error (*n* = 4). Letters within each bar graph **(D–F)** indicate significant differences as determined by Kruskal–Wallis and Bonferroni *post hoc* testing.

Concurrent with NO_3_^–^ consumption in the slurries, N_2_O production increased rapidly between 24 and 48 h, especially in treatments without added Cu (Figure 2A). The control and treatments with Mo, Fe, and Mo + Fe accumulated significantly more N_2_O (approximately 79, 55, 62, and 94% of added NO_3_^–^, respectively), than the treatments with Cu, Fe + Cu, Mo + Cu, and Mo + Fe + Cu (approximately 13, 14, 4, and 11% of the added NO_3_^–^, respectively; Figure 2D). Total N_2_O concentrations reached a plateau between 48 and 96 h, most likely due to exhaustion of available C and NO_3_^–^. CH_4_ accumulated quickly in the microcosms (6–12 h) and continued to be produced for the duration of the incubation, reaching a final concentration of ∼0.8 mmoles (Figure 2B). By the end of the incubation, all metal additions caused an increase in CH_4_ production relative to the control, though the differences were only significant between the control and the Cu, Mo + Cu, and Mo + Fe + Cu treatments (Figure 2E). CO_2_ production also increased throughout the incubation period, most rapidly between 12 and 24 h, and reached ∼2 mmoles CO_2_ at the end of the incubation (Figure 2C). As with CH_4_, the only significant treatment differences for CO_2_ production were between the control and the Cu, Mo + Cu, Mo + Fe + Cu treatments (Figure 2F).

### The Effects of Trace Metal Addition on Targeted Microbial Groups

**FIGURE 3:**
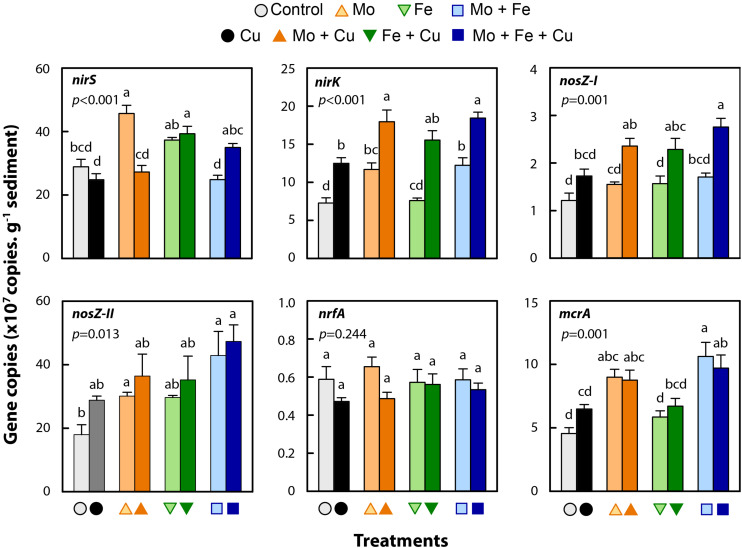
Gene copies per g sediment (dry weight) at the end of incubation (96 h) in microcosms amended with molybdate (Mo), iron (Fe), copper (Cu), and combinations thereof. Genes were targeted representing nitrite reducers (*nirS*, *nirK*, and *nrfA*), nitrous oxide reducers (*nosZ-I* and *II*), and methanogens (*mcrA*). Data are shown as mean ± standard error (*n* = 4). Letters within each bar graph indicate significant differences as determined by Kruskal–Wallis and Bonferroni *post hoc* testing.

Bacterial abundance in the control treatment was 5.4 × 10^8^ (±4.5 × 10^7^) *16S* rDNA copies g^–1^ sediment dry weight, which did not differ significantly from the abundance estimates obtained for the Cu, Mo, Mo + Fe, or Mo + Fe + Cu treatments. Abundance in the three remaining treatments (Fe, Fe + Cu, and Mo + Cu) was slightly higher (1.4-fold) and significantly different from the control (*p* = 0.005; Supplementary Figure S1). Trace metal addition affected the abundance of *nirK* (*p* < 0.001), *nirS* (*p* < 0.001), *nosZ-I* (*p* = 0.001) and *nosZ-II* (*p* = 0.013) denitrifying microbial groups (Figure 3). Reducers of NO_2_^–^ utilizing Cu-NIR (*nirK*) were more abundant in all treatments when compared to the control, though the increase due to Fe addition was not significant. Microbial groups having a cytochrome *cd*_1_-NIR (*nirS*) were relatively more abundant in the Mo and Fe + Cu treatments. The *nosZ* clade I and II microbial groups responsible for the reduction of N_2_O to N_2_ were in general more abundant in the treatments that received Cu. We observed no effect of trace metals on DNRA NO_2_^–^ reducers (*nrfA*, *p* = 0.24; Figure 3) but found that methanogens were more abundant in the treatments containing additional Mo (Mo, Mo + Cu, Mo + Fe, and Mo + Fe + Cu; *mcrA* (*p* = 0.001); Figure 3) when compared to the control.

## Discussion

**FIGURE 4:**
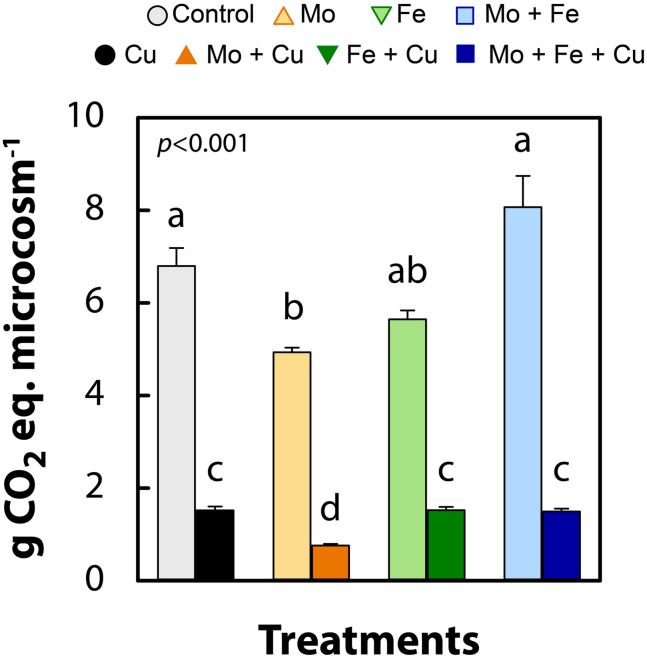
Global warming potential in g of CO_2_ equivalents (g CO_2_-eq) of cumulative gas concentrations (96 h) of nitrous oxide, methane, and carbon dioxide in anoxic slurries amended with molybdate (Mo), iron (Fe), copper (Cu), and combinations thereof. Data are shown as mean ± standard error (*n* = 4). Letters indicate significant differences as determined by Kruskal–Wallis and Bonferroni *post hoc* testing.

We observed that trace metal (Mo, Fe, Cu, and their combinations) addition to wetland sediments regulated GHG emissions (Figure 2) and altered the abundance of microbial functional groups typically associated with N removal and C cycling (Figure 3). Previous studies have examined the effects of trace metal availability on these processes, often reporting inhibitory effects (Giller et al., 1998; Magalhães et al., 2007, 2011; Deng et al., 2018; Keller and Wade, 2018) however, the range of metal concentrations previously tested far exceeds the levels applied in the current study. The key interpretation of the current experiment is that when available at trace levels, metals, specifically Cu, enhanced the reduction of N_2_O to N_2_ and increased CH_4_ and CO_2_ emissions from wetland sediments. When N_2_O, CH_4_, and CO_2_ were combined and expressed as g CO_2_-eq per microcosm, all treatments receiving Cu addition (Cu, Mo + Cu, Fe + Cu, and Mo + Fe + Cu) had significantly lower CO_2_-eq emissions (*p* < 0.001; Figure 4), indicating a major role of Cu in anaerobic respiration (denitrification and methanogenesis) as a regulator of nutrient cycling and GHG emissions.

### Denitrification

In our experiments, none of the trace metal additions had an effect on NO_3_^–^ removal (Figure 1), which is somewhat surprising since all bacterial nitrate reductases contain a Mo cofactor at their active sites (Moreno-Vivian et al., 1999). This may indicate that Mo was not limiting to the denitrifiers in this set of soils. Also, we observed a small delay in NO_3_^–^ reduction (∼24 h) in all treatments that could be attributed to the lag time required to induce the denitrifying pathway within a population less accustomed to substantial concentrations of this alternative electron acceptor (Dodla et al., 2008). Concurrent with the loss of NO_3_^–^, we observed minor transient accumulation of NO_2_^–^; this pattern has been observed frequently (Cooper and Smith, 1963; Taghizadeh-Toosi et al., 2020; Wang et al., 2020) and is thought to be a result of stochastic transcriptional regulation of the *nir* operon (Hassan et al., 2016). Following NO_2_^–^ reduction, NO is produced. Though we did not analyze NO concentrations, it is unlikely that NO accumulated in our microcosms but was instead quickly reduced to N_2_O. Since NO has potent cytotoxicity at μM levels (Chaudhari et al., 2017; Hartop et al., 2017), any accumulation would have had a negative effect on other aspects of microbial metabolism, particularly CO_2_ production, which we did not observe.

Regardless of what other metals were included in the microcosms, we observed a dramatic effect of Cu addition, leading to enhanced reduction of N_2_O to N_2_ (Figure 2) and an increased abundance of nitrite (*nirK*) and nitrous oxide (*nosZ* clade *I* and *II*) reductase genes (Figure 3), which both code for Cu-containing enzymes. Overall, gene abundance for *nosZ* clade II was ∼10-fold greater than for clade I, which is consistent with recent reports that clade II is the dominant form in many soil ecosystems (Jones et al., 2013, 2014; Orellana et al., 2014; Ligi et al., 2015; Graves et al., 2016). Clade II is also affiliated with a broader diversity of organisms than Clade I including several non-denitrifiers (Graf et al., 2014; Hallin et al., 2018), so it is important to recognize that our Clade II estimates may reflect changes in community composition that do not necessarily directly relate to N_2_O reduction. Subsequent analysis using primers that target subclades within Clade II (*sensu* Chee-Sanford et al., 2020) or metagenomic sequencing could help resolve this and provide a better understanding of the ecophysiology of various *nosZ* populations. The overall strong effect of Cu on N_2_O reduction was prevalent because the last step of denitrification is catalyzed by a Cu-containing reductase and no alternative pathways for N_2_O transformation have yet been discovered (Thomson et al., 2012). In other laboratory experiments, Cu has similarly been found to be a strong driver of denitrifying metabolism. For example, Cu-deficient cells can have more transcripts of *nosZ* and Cu-scavenging genes to compensate for the loss of N_2_O reduction due to limited Cu availability (Felgate et al., 2012). Also, strains deficient in Cu-transporters and chaperones may be unable to reduce N_2_O to N_2_, forming a nonfunctional NOR, even though *nosZ* is expressed (Sullivan et al., 2013). In our microcosms, denitrifying bacteria were able to rapidly take advantage of excess NO_3_^–^ due to copious bioavailable Cu for synthesis of NOS. Even though concentrations of NO_3_^–^ and Cu are usually lower in environmental scenarios, similar trade-offs may exist, which could affect ecosystem emissions of N_2_O and require further investigation.

### DNRA

Over the course of the study, small accumulations of pore-water NH${}_{4}^{+}$ occurred (Figure 1) depending on the type of metal added, and DNRA could be another possible factor affecting the fate of added NO_3_^–^. The accumulation of NH_4_^+^ was greatest in treatments that contained both Mo and Fe (i.e., Mo + Fe and Mo + Fe + Cu), which may indicate a synergistic response of the enzymes that reduce NO_3_^–^ (all nitrate reductases are Mo-dependent) and the DNRA-specific nitrite reductase (*nrfA*), which includes a multi-heme complex. Overall, the accumulated NH_4_^+^ (<1 mM) was much lower than the amount of NO_3_^–^ removed (∼10 mM), and the abundance of the DNRA marker gene *nrfA* was low (Figure 3), so it is likely that negligible DNRA occurred in our microcosms. Indeed, anoxic organic matter mineralization (as indicated by consistent CO_2_ production) could contribute to the accumulation of NH4+ and the observed differences due to metal addition could be indirect responses. DNRA and N mineralization commonly co-occur in wetland sediments (White and Reddy, 2009), but DNRA seems to be important mainly in environments with high C availability and limited NO_3_^–^ availability (Hill, 2019). More conclusive results on DNRA activity in the present wetland setting require additional analyses, e.g., using ^15^NO_3_^–^ to trace the production of ^15^NH_4_^+^ from DNRA. Additional analyses using alternate *nrfA* primers could also be informative. In particular, the qPCR primers we used have poor coverage of NrfA Clade I and thus limited ability to detect members of the family *Geobacteraceae*, which have recently been found to be potentially significant for DNRA across a range of different soil habitats (Nelson et al., 2016). Given the recognized importance of *Geobacteraceae* in metal cycling (Röling, 2014), further consideration of this group using novel primers such as those recently developed by Cannon et al. (2019) would better assess the genetic potential for DNRA and effects of trace metal availability.

### Effect on Carbon Mineralization

Wetland soils are large C sinks because anoxic conditions constrain organic matter mineralization and oxidation. In freshwater wetlands, the lack of electron acceptors such as O_2_, NO_3_^–^, and SO_4_^2–^, may lead to fermentative and reductive conditions where organic C will be reduced to CH_4_ (Kim et al., 2015; Cheng et al., 2019). In this study, the pattern of CO_2_ production primarily reflected the magnitude of denitrification, as discussed earlier. We assumed that the fraction of total CO_2_ as bicarbonate (HCO^–^) was likely constant, because at the end of the incubation pH increased approximately 1 pH-unit due to denitrification and no substantial differences were found in pH among the treatments (Supplementary Table S2). Equally, we did not quantify and identify forms of dissolved organic carbon as this was out of the scope of this study. We hypothesized that denitrifier activity would outcompete methanogen activity similar to the findings of Klüber and Conrad (1998); rather, CH_4_ accumulated throughout the incubations (Figure 2B). This result was unexpected because denitrification should suppress methanogenesis as a result of the higher Gibb’s free energy (ΔG^0^). This set of sediments originates from a freshwater wetland that is known to produce significant amounts of CH_4_ and host diverse methanogenic communities (Morrissey and Franklin, 2015; Berrier, 2019; Dang et al., 2019). It appears that the inhibitory effect of NO_3_^–^ addition was transient or partial. It is possible that the rapid NO_3_^–^ consumption and subsequent removal of denitrification intermediates alleviated inhibitory effects on methanogenesis. Since both processes were occurring, it is evident that denitrifiers and methanogens were competing for available C.

Overall, the higher abundance of methanogens (*mcrA*) and noticeable accumulation of CH_4_ in all metal treatments versus the control indicates an important role of trace metal availability in CH_4_ cycling in our system. As expected, Mo addition enhanced the abundance of *mcrA* genes (Mo, Mo + Cu, Mo + Fe, and Mo + Fe + Cu treatments; Figure 3). This is because a Mo co-factor is typically required for formylmethanofuran dehydrogenase (*fmd*) that catalyzes the reduction of CO_2_ to formyl-methanofuran in the first step of methanogenesis by reduction of CO_2_ with electrons from H_2_ (Glass and Orphan, 2012). The subsequent step in methanogenesis is catalyzed by MCR, which is a Ni-containing enzyme. Because we did not add Ni in any of our treatments, we assumed that MCR would not change (or would decrease due to competition with denitrification). Instead, Mo addition and the increasing partial pressure of CO_2_ in the bottles during the incubation seem to have triggered a shift toward a Mo-based methanogenesis pathway. In general, CH_4_ production in Pamunkey River sediments have been found to proceed by hydrogenotrophic, acetoclastic, and syntrophic pathways (Berrier, 2019; Dang et al., 2019).

Though no Cu-dependent enzymes have yet been identified in the methanogenesis pathways (Glass and Orphan, 2012), the effect of Cu concentration on CH_4_ emissions from wetlands is an area of active research. Prior work by Keller and Wade (2018) found a strong suppression of CH_4_ emissions following Cu amendments to peatland soils, and argued broadly that Cu may inhibit methanogenesis in wetland environments. This contradicts our finding that Cu addition elicited more CH_4_ than the control (Figure 2), as well as similar results from Thomas and Pearce (2004). Interestingly, the discrepancy across these three studies cannot be explained by simply looking at the concentration of Cu added; our Cu amendments were approximately 10-fold lower than those made by Keller and Wade (2018), whereas the amendments made by Thomas and Pearce (2004) were 10-fold higher. Understanding the effects of Cu on net CH_4_ fluxes requires further study and should consider potential mitigating factors such as soil texture and cation exchange capacity (Thomas and Pearce, 2004), the impact of Cu amendments on dissolved organic carbon availability (Jiao et al., 2005), and metal effects on methane oxidation (Mohanty et al., 2000).

### Environmental Implications

Previous studies have examined the effects of trace metal abundance (or addition) on N and C cycling in sediments, peat, and agricultural soils. Typically, those studies report an inhibitory effect; however, the ranges of trace metal concentrations in those studies far exceed the levels of the current study. For instance, Keller and Wade (2018) amended peat slurries with ∼200 μM trace element solution and found a significant decrease in CH_4_ emission, most likely due to Cu-induced toxicity. Similarly, Magalhães et al. (2007) observed 85% inhibition of denitrification accompanied by accumulation of substantial N_2_O and NO_2_^–^ at 79 μg Cu g^–1^ sediment (equivalent to 1200 μM Cu) in estuaries. In a follow-up study, lower denitrification rates due to Cu (∼900 μM) were accompanied by a decline in the abundance and β-diversity associated with the *nirK*, *nirS*, and *nosZ* microbial groups (Magalhães et al., 2011), while keeping in mind that the coverage of those primers has improved greatly since then. Elsewhere, the particularly low concentrations of dissolved Cu, Fe, and Mo (∼5, 1, and 0.2 μg L^–1^, respectively) and the copious dissolved organic C (∼80 mg L^–1^) (Raudina et al., 2017) in peatlands triggered substantial emissions of CH_4_ and N_2_O (Basiliko and Yavitt, 2001; Basiliko et al., 2013; Voigt et al., 2017). A more recent study that tested the effects of Cu pollution (addition of 100 μg L^–1^, ∼1.6 μM) on urban freshwater wetland sediments found significantly reduced CH_4_ emissions, but no change in N_2_O emissions (Doroski et al., 2019). In the latter case, the lack of a N_2_O response could be due to low NO_3_^–^ availability (18 μM) relative to C.

The abundance of bioavailable metals in the environment, typically in their ion forms, is often several orders of magnitude lower than the routinely reported total metal content. Further, the ion form of trace metals could be unavailable to microorganisms due to physicochemical interactions (release and sorption kinetics with soil particles and organic matter) and plant uptake (Giller et al., 1998). At neutral and alkaline pH levels, metals tend to be effectively immobilized as inorganic compounds (metal-oxides, -hydroxides, and -carbonates). Additionally, organic complexes such as humic ligands are known to bind metal cations thus lowering their availability to microbes and plants. Furthermore, hydrogen sulfide (H_2_S), naturally produced due to dissimilatory SO_4_^2–^ reduction in salt marshes, may limit the availability of trace metals through the production of insoluble metal-sulfide complexes with potential implications for N cycling and N_2_O emissions (Gauci et al., 2004; Butterbach-Bahl et al., 2013). Sulfate-reducing bacteria may outcompete methanogens and thus suppress CH_4_ emissions in salt marshes. In the current study, the added SO_4_^2–^ as CuSO_4_ and FeSO_4_ could potentially have been reduced to H_2_S; however, the initial and final porewater SO_4_^2–^ concentrations remained at comparable levels (average change over time across treatments was only 2.8%, Supplementary Table S2) and no sulfide odor was detected when microcosms were opened.

Out of the three metals tested here, Fe^2+^ addition does not appear to influence N and C cycling in this set of sediments. Fe^3+^ is an abundant element in our biosphere including wetlands, however not directly biologically available, as it has to be reduced to Fe^2+^. Fe^2+^ was added in our microcosms due to its role in the reduction of NO_2_^–^ to NO with cytochrome *cd1* NIR. Apparently, this set of sediments has the capacity to supply enough Fe^2+^. Another, Fe-dependent N-cycling process besides denitrification and DNRA is anaerobic ammonium oxidation (anammox), which relies heavily on cytochromes and oxyreductases containing Fe (Ferousi et al., 2017), and its relative prevalence could be determined by isotopic assessments (Brunner et al., 2013).

*Ex situ* experimentation offers key advantages in disentangling factors in a controlled environment. It is acknowledged that longer incubation times could shift the microbial community toward a fitter structure for trace metal or high levels of NO_3_^–^ utilization. The aim of this study was to investigate the prospective of trace metal additions stimulating mixed-community microbial systems, as seen previously on model organisms. Equally, longer incubations, frequent sampling and non-limiting substrates could help us illustrate the potential of trace metals to enhance microbial functioning and microbial groups. Follow-up studies should consider shorter incubation times and lower substrate concentrations to understand the temporal shift in microbial function and diversity upon trace metal additions.

The present results suggest that trace levels of Cu exert a more important effect on N_2_ and N_2_O emissions from the environment than previously thought. These findings are important not only for understanding fundamental controls on N_2_O production, but also for making predictions about potential future emissions given increasing nutrient and metal loads associated with urban pollution. Likewise, the effect of trace metal availability on denitrification enzymology also is of considerable interest in the context of agriculture and food production, and it has been suggested that metal additions could be a possible strategy to mitigate high soil N_2_O emissions associated with crop production (Richardson et al., 2009; Shen et al., 2019).

## Conclusions and Future Challenges

Our results demonstrate that trace metal availability affects microbial processes, resulting in greater GHG emissions and C mineralization. We found that even short-term (96 h) manipulation of trace metal availability led to changes in community structure (functional group abundance) and potential increases in GHG emissions. In particular, our results suggest that trace metal bioavailability may, directly or indirectly, regulate or co-limit denitrification kinetics and favor more active microbial communities that are able to quickly acquire the bioavailable metals and incorporate them in functional oxidoreductases. Disentangling the potential controls of various metals on denitrification and other N-cycling processes such as DNRA and anaerobic ammonium oxidation (anammox) and co-varying effects on C cycling, requires additional experimentation across a range of metal concentrations, substrates [e.g., lower (NO_3_^–^)] and biochemical processes in different soil types and ecosystems. Future studies should also investigate metagenomic and metatranscriptomic profiles to understand and evaluate the ecological importance of trace metal limitation. This would allow a more comprehensive examination of potential impacts beyond the small group of functional genes that we considered, and would avoid issues such as primer bias, poor coverage, and inefficient amplification often associated with qPCR of phylogenetically diverse groups. Such studies should be accompanied by high-throughput metabolite or process rate analysis either in the lab or in the field.

## Data Availability Statement

All datasets presented in this study are included in the article/Supplementary Material.

## Author Contributions

GG designed the experiments, performed lab analysis, statistically tested and interpreted the data, and wrote and reviewed the manuscript. KRH performed lab analysis, and wrote and reviewed the manuscript. BLB, BS, and LE performed lab analysis and reviewed the manuscript. RBF performed lab analysis, interpreted data, and wrote and reviewed the manuscript. All authors contributed to the article and approved the submitted version.

## Conflict of Interest

The authors declare that the research was conducted in the absence of any commercial or financial relationships that could be construed as a potential conflict of interest.

## Supplementary Material

FIGURE S1 Average *eub* gene copies g^–1^ sediment (dry weight) at the end of incubation (96 h) in microcosms amended with molybdate (Mo), iron (Fe), copper (Cu), and combinations thereof. Letters above each bar graph indicate significant differences as determined by Kruskal–Wallis and Bonferroni *post hoc* testing.

TABLE S1 Growth (OD_600_) and headspace N_2_O (ppm) at 24 h for *Paracoccus denitrificans* incubated at different metal levels, under denitrifying conditions, in denitrifying mineral medium containing 20 mM NO_3_^–^. Cu^2+^, total Mo and total Fe were determined by ICP-OES at Environmental Sciences Analytical Labs, School of Environmental Sciences, University of East Anglia, United Kingdom (Unpublished data, Ph.D. Thesis, Giannopoulos, G. 2015).

TABLE S2 Initial (0 h) and final (96 h) SO_4_^2–^ concentrations and pH (96 h) of microcosm pore-water in the various treatments (*n* = 4, mean ± SE).
