# Supplementary figures and images for: Trace Metal Availability Affects Greenhouse Gas Emissions and Microbial Functional Group Abundance in Freshwater Wetland Sediments
Source: Front Microbiol. 2020 Sep 30;11:560861. doi: 10.3389/fmicb.2020.560861 (PMC7561414; doi:10.3389/fmicb.2020.560861)

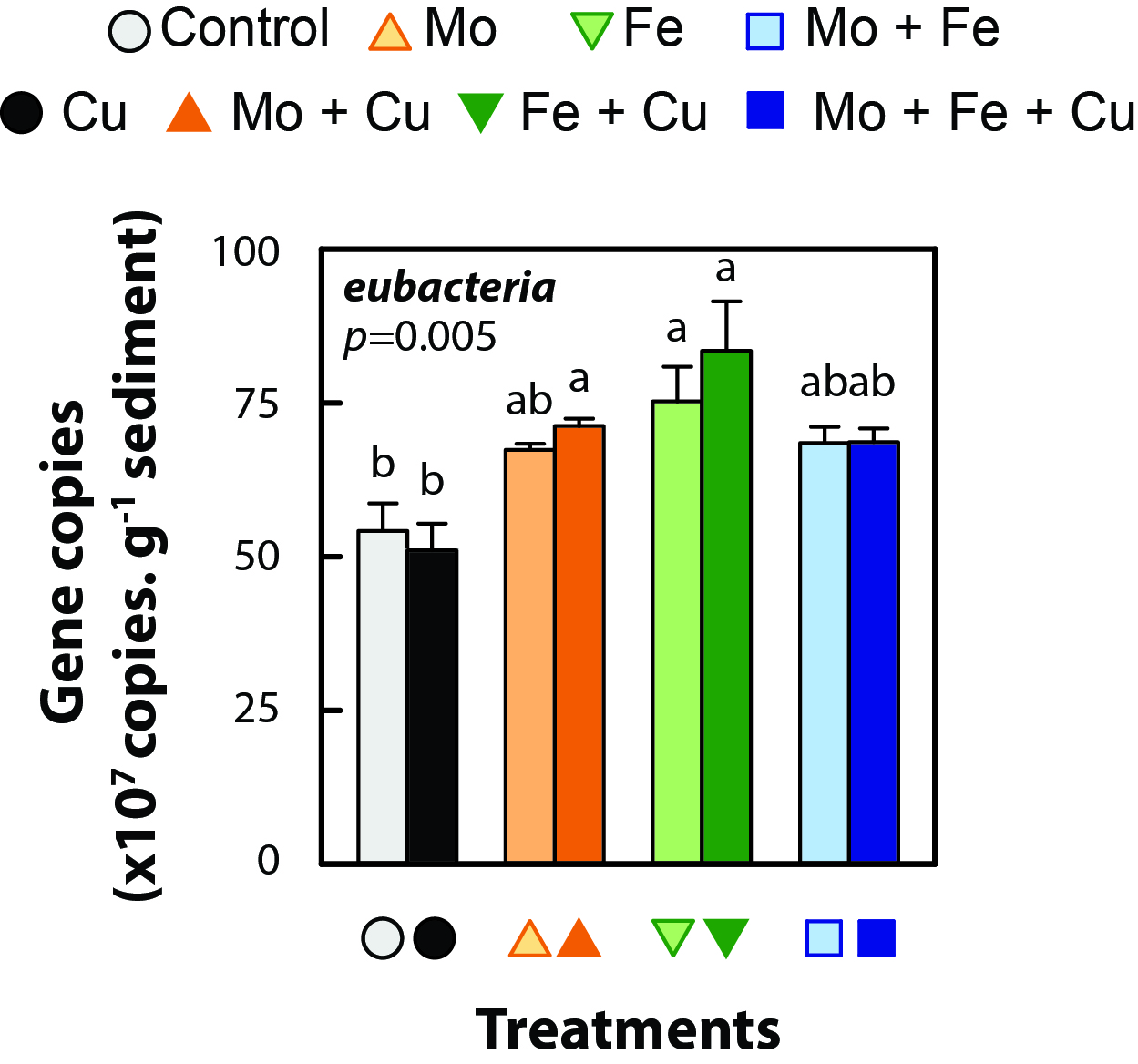

Supplement: FIGURE S1 — Average eub gene copies g–1 sediment (dry weight) at the end of incubation (96 h) in microcosms amended with molybdate (Mo), iron (Fe), copper (Cu), and combinations thereof. Letters above each bar graph indicate significant differences as determined by Kruskal–Wallis and Bonferroni post hoc testing. [file Image_1.JPEG]
