# Supplementary material for: Trace Metal Availability Affects Greenhouse Gas Emissions and Microbial Functional Group Abundance in Freshwater Wetland Sediments
Source: Front Microbiol. 2020 Sep 30;11:560861. doi: 10.3389/fmicb.2020.560861 (PMC7561414; doi:10.3389/fmicb.2020.560861)
Supplement: TABLE S1 — Growth (OD600) and headspace N2O (ppm) at 24 h for Paracoccus denitrificans incubated at different metal levels, under denitrifying conditions, in denitrifying mineral medium containing 20 mM NO3–. Cu2+, total Mo and total Fe were determined by ICP-OES at Environmental Sciences Analytical Labs, School of Environmental Sciences, University of East Anglia, United Kingdom (Unpublished data, Ph.D. Thesis, Giannopoulos, G. 2015). [file Table_1.pdf]

Supplementary Material – Table S1

Georgios Giannopoulos[1, 2]*, Katherine R. Hartop[1, 3], Bonnie L. Brown[4], Bongkeun Song[5], Lars Elsgaard[6] and Rima Franklin[1]

Trace metal availability affects greenhouse gas emissions and microbial functional group abundance in freshwater wetland sediments.

Front. Microbiol. | doi: 10.3389/fmicb.2020.560861

[1]Department of Biology, Virginia Commonwealth University, United States

[2]School of Agriculture, Aristotle University of Thessaloniki, Greece

[3]Royal Society of Chemistry, United Kingdom

[4]College of Life Sciences and Agriculture, University of New Hampshire, United States

[5]Department of Biological Sciences, William & Mary's Virginia Institute of Marine Science, College of William & Mary, United States

[6]Department of Agroecology - Soil Fertility, Aarhus University, Denmark

Table S1. Growth (OD $_{600}$) and headspace N_2O (ppm) at 24 h for *Paracoccus denitrificans* incubated at different metal levels, under denitrifying conditions, in denitrifying mineral medium containing 20 mM NO_3^-. Cu^{2+}, total Mo and total Fe were determined by ICP-OES at Environmental Sciences Analytical Labs, School of Environmental Sciences, University of East Anglia, UK (Unpublished data, PhD Thesis, Giannopoulos, G. 2015).

**Cu^{2+} (μM)**	**Mo (μM)**	**Fe (μM)**	**OD $_{600}$ (24 h)**	**Headspace N_2O (ppm)**
0.32	0.35	0.91	0.73	5841
0.30	0.32	0.78	0.75	5589
1.07	1.15	3.05	0.78	67
1.15	1.17	3.32	0.8	67
4.05	4.38	11.75	0.77	67
3.59	4.20	10.02	0.75	68
9.52	10.20	27.20	0.64	76
8.72	9.42	24.58	0.69	76
12.32	13.20	35.20	0.71	71
12.08	12.94	34.54	0.78	71
16.56	17.90	46.96	0.75	67
15.78	16.74	45.09	0.71	67
23.59	25.63	67.40	0.72	72
24.83	26.05	70.94	0.7	72
28.02	29.85	81.29	0.52	2799
29.16	31.33	83.57	0.55	3041
48.71	52.36	140.57	0.35	5481
50.05	53.10	143.00	0.42	5416
