# Supplementary material for: Trace Metal Availability Affects Greenhouse Gas Emissions and Microbial Functional Group Abundance in Freshwater Wetland Sediments
Source: Front Microbiol. 2020 Sep 30;11:560861. doi: 10.3389/fmicb.2020.560861 (PMC7561414; doi:10.3389/fmicb.2020.560861)
Supplement: TABLE S2 — Initial (0 h) and final (96 h) SO42– concentrations and pH (96 h) of microcosm pore-water in the various treatments (n = 4, mean ± SE). [file Table_2.pdf]

Supplementary Material – Table S2

Georgios Giannopoulos[1, 2]*, Katherine R. Hartop[1, 3], Bonnie L. Brown[4], Bongkeun Song[5], Lars Elsgaard[6] and Rima Franklin[1]

Trace metal availability affects greenhouse gas emissions and microbial functional group abundance in freshwater wetland sediments.

Front. Microbiol. | doi: 10.3389/fmicb.2020.560861

[1]Department of Biology, Virginia Commonwealth University, United States

[2]School of Agriculture, Aristotle University of Thessaloniki, Greece

[3]Royal Society of Chemistry, United Kingdom

[4]College of Life Sciences and Agriculture, University of New Hampshire, United States

[5]Department of Biological Sciences, William & Mary's Virginia Institute of Marine Science, College of William & Mary, United States

[6]Department of Agroecology - Soil Fertility, Aarhus University, Denmark

Table S2 Initial (0 h) and final (96 h) SO_4^{2-} concentrations and pH (96 h) of microcosm pore-water in the various treatments (*n* = 4, mean ± SE).

Treatment	Initial [SO_4^{2-}] µM	Final [SO_4^{2-}] µM	Final pH
Control	4.9 ± 0.8	8.5 ± 3.5	7.6 ± 0.2
Cu	23.4 ± 2.9	20.3 ± 2.0	7.8 ± 0.2
Fe	68.9 ± 2.3	57.0 ± 8.4	7.7 ± 0.2
Fe+Cu	99.2 ± 6.3	78.0 ± 33.3	7.7 ± 0.2
Mo	15.0 ± 3.8	17.5 ± 2.7	7.8 ± 0.2
Mo+Cu	21.4 ± 0.9	25.4 ± 2.7	7.9 ± 0.2
Mo+Fe	68.1 ± 3.9	63.5 ± 5.0	7.7 ± 0.2
Mo+Fe+Cu	133.0 ± 18.0	138.3 ± 21.0	7.9 ± 0.2
